# Visa Status, Physical Activity and Mental Health Among Farsi/Dari Speaking Refugees, Immigrants and Asylum Seekers in Sydney, Australia

**DOI:** 10.1002/hpja.70032

**Published:** 2025-04-03

**Authors:** Reza Rostami, Ruth Wells, Jila Solaimani, David Berle, Derrick Silove, Simon Rosenbaum, Zachary Steel

**Affiliations:** ^1^ Psychiatry & Mental Health, School of Clinical Medicine University of New South Wales Sydney Australia; ^2^ Clinical Psychology, Graduate School of Health University of Technology Sydney Sydney Australia; ^3^ St John of God Health Care North Richmond Hospital North Richmond Australia

## Abstract

**Background:**

Asylum‐seekers in Australia are subjected to restrictive access, such as to education, work, and family reunion, which can affect mental and physical health. We examined the relationship between these restrictive measures and mental health symptoms and physical activity in a sample of Iranian and Afghan asylum seekers, refugees, and immigrants in Sydney.

**Methods:**

276 Iranian and Afghan asylum seekers, refugees, and immigrants were recruited using a probability proportional to size representative, time by location sampling frame across randomly selected Iranian and Afghan grocery shops in Sydney. The interview recorded physical activity levels (Simple Physical Activity Questionnaire); posttraumatic stress disorder (PTSD) symptoms and trauma events (Harvard Trauma Questionnaire); depression symptoms (Hopkins Symptom Checklist) and demographics (Visa, gender, age). Cross‐sectional hierarchical logistic regression examined the relationship between visa status and achieving World Health Organisation physical activity guidelines. Hierarchical linear regression examined visa status and sedentary time. Path Analysis tested whether these relationships were mediated by PTSD or depression symptoms.

**Results:**

Forty‐six percent of Iranian and Afghan respondents who had secure residency in Australia engaged in levels of moderate to vigorous activity that met the WHO recommended rates. After controlling for variables, asylum seekers (with insecure visas) were three times less likely to meet guidelines and reported an average of 30 min per day more sedentary time. Mediation analysis indicated that depression symptoms and PTSD symptoms mediated the visa status and physical activity relationship. Likewise, depression and PTSD mediated the relationship between visa status and sedentary behaviour.

**Conclusion:**

Insecure visa status is associated with physical inactivity and sedentary behaviour, which are influenced by psychiatric symptoms. This could have long‐term physical and mental health consequences for asylum seekers.

**So, What?:**

Visa restrictions placed on people seeking asylum when they arrive in Australia could have long‐term physical and mental health consequences. This could affect the quality of life for those affected, as well as placing a greater burden on the health system in the future.

## 1 Introduction

There are currently over 110 million people living in forced displacement globally. The United Nations High Commission for Refugees (UNHCR) predicts that the need for refugee resettlement in 2024 will grow by 20% compared to 2023 [1]. A small minority are resettled in countries like Australia, where asylum seekers live with insecure residency and the threat of being sent back to situations of danger while waiting for their refugee claims to be assessed. Refugee visas, however, provide increased residency security and greater access to services. In 2021, there were about 3.1 million adults and children awaiting a decision on their protection claims within high‐income countries [2]. In August 2012, the Australian Government suspended the processing of refugee claims for people arriving by boat, leaving 30 500 asylum seekers living in Australia for prolonged periods without determination of their claims [3].

Asylum seekers and refugees are exposed to numerous risk factors for psychological disturbance. This includes exposure to violence, forced displacement, and multiple losses [4, 5, 6]. Asylum seekers, who face the additional challenge of insecure residency, are at heightened risk of more severe and prolonged mental health problems [7]. Research in Australia has shown how children in families from refugee backgrounds reported similar levels of psychosocial functioning to children in the general Australian population. This demonstrates how resettled refugee families can flourish when given security and access to the same services as Australian citizens [8]. However, people who arrived in Australia by boat and sought political asylum are subjected to a number of restrictions, such as restricted access to family reunion, legal support, interpreting services, and settlement support services [3]. Children in families seeking asylum reported approximately double the level of psychosocial problems when compared with families with secure refugee status [9]. While evidence of the association between asylum policies which prolong uncertainty and mental health problems has been established [10], the additional impact of these policies on modifiable physical health indicators and lifestyle behaviours is yet to be demonstrated.

Physical inactivity is associated with an increased risk of all‐cause morbidity and mortality [11], and is therefore an early indicator of future quality of life and life expectancy. The link between physical activity and mental health is well established [12], as is the effectiveness of physical activity interventions for improving mental health [13]. Physical activity is a powerful tool in promoting mental health among different populations [14]. It is associated with reduced levels of stress, anxiety, and depression while increasing self‐esteem and overall well‐being. However, research is needed to understand the impact of resettlement stressors on physical activity and mental health [15]. The unique circumstances of refugees, such as limited access to resources and social support networks, may affect their ability to engage in physical activity as a means of coping [16]. Additionally, for individuals with insecure visa statuses, access to physical activity can be significantly constrained by the challenges associated with visa restrictions. This limitation not only impacts their physical well‐being but also their mental health, creating a brutal cycle that may further inhibit their ability to engage in healthy behaviours.

The impact of refugee and asylum stresses needs to also be understood against the background of broad cultural and social patterns of physical activity. The current research is focused on the impact of refugee and asylum stress on Iranian and Afghan communities that have arrived in Sydney, Australia since 2010. The Iranian and Afghan populations have diverse cultural, linguistic, and social backgrounds shaped by their distinct histories and regional influences which are associated with different patterns of physical activity in these communities [17]. Iran is characterised by a relatively urban society, with widespread access to education, health services, and cultural traditions that support physical activities such as wrestling, soccer, and hiking. Persian (Farsi) is the dominant language, and the country has a rich art, literature, and sports heritage [18]. Afghanistan, in contrast, has until very recently been predominantly rural, with a multi‐ethnic population that includes Pashtuns, Tajiks, Hazaras, and Uzbeks. Dari (a dialect of Persian) and Pashto are the main languages, and traditional activities such as Buzkashi reflect the country's nomadic and tribal roots [19]. Both populations face unique challenges as refugees and asylum seekers, often resulting from political instability, war, and socio‐economic hardship in their home countries [20]. Afghan refugees are more likely to have experienced severe disruptions in education and healthcare, while Iranians may be more exposed to urban lifestyles and organised sports [21]. Despite these differences, both groups face barriers to integration, including language barriers, mental health issues, and limited access to culturally sensitive programmes in host countries, which can affect overall well‐being and physical activity levels.

Additionally, gender norms play a crucial role, with Iranian women often experiencing more freedom to engage in physical activity, especially in gender‐segregated environments, compared to Afghan women, who face stricter societal restrictions [22]. Religious practises, including modesty requirements and prioritisation of spiritual obligations, also shape attitudes towards PA [23].

Cultural practises and societal norms also play a significant role in influencing patterns of physical activity among populations and community groups [24]. For example, traditional Iranian and Afghan gender roles often limit women's participation in outdoor or mixed‐gender activities, while men may prioritise work and family responsibilities over structured exercise [25]. Strong family and community bonds mean that activities involving these groups, such as walking or traditional dances, are more culturally appropriate than individual workouts. Religious considerations, like modesty and prayer schedules, further influence activity choices, and traditional practises like wrestling or walking during pilgrimages contribute to fitness in culturally specific ways [26]. Consideration of Physical activity requires a broad definition, including planned exercises (e.g., football, swimming, running) and incidental activities (e.g., walking to the market or housework). Taking this broader definition, population‐based studies from Iran have indicated that regular engagement in physical activity is reported by just under 50% of the general population, with females reporting lower rates (42%) than males (58%). Higher physical activity was associated with higher socio‐economic status, living in rural settings, and being single. Reduced engagement in recreational activities was found to be a major continuing factor leading to reduced physical activity levels. There has been comparatively less research among general Afghan populations; using the IPAQ self‐report measure of physical activity, 64% of men and 58% of women reported regular physical activity, with some similar patterns to Iran, in that PA was greater in men (63.9%) than in women (57.6%) was greater among single participants and literate individuals, a measure of wealth in this context.

There is limited research that has examined the extent to which these levels of physical activity are retained among refugee and immigrant communities once the settle in to a host country. Barriers such as a lack of culturally inclusive spaces, language challenges, financial constraints, and prioritisation of survival needs often hinder participation [27]. To address this, culturally sensitive strategies are crucial, including gender‐specific programmes, incorporation of traditional exercises, community engagement, and education in native languages. Recognising these cultural norms and practises can help in tailoring physical activity programmes that feel familiar and inclusive, while addressing potential barriers such as gender roles, modesty, and access to culturally appropriate facilities [24, 28].

Although several studies [29, 30, 31, 32] have explored the association between refugees and physical activity or the effectiveness of physical activity interventions on psychological outcomes, it is noteworthy that there is a significant gap in research focusing on this aspect in the context of different visa statuses in Australia and other countries. We, therefore, sought to understand this relationship. In a representative sample of Dari and Farsi speaking refugees and asylum seekers and immigrants from Iran and Afghanistan in Sydney, we aimed to:

1. Determine the association between visa status (secure/insecure) and whether participants meet the WHO physical activity guidelines.
2. Determine the association between visa status and levels of sedentary behaviour.
3. Determine the association between visa status and the odds of meeting the physical activity guidelines or levels of sedentary behaviour after controlling for age, gender, country, and previous trauma history.
4. Determine whether this relationship is mediated by psychiatric symptoms (depression or PTSD).

We hypothesised that, compared to participants with secure visas, people with insecure visa status would be less likely to meet the physical activity guidelines and report more sedentary behaviour, and that this relationship would be mediated by psychiatric symptom severity.

## 2 Methods

### 2.1 Study Design and Participants

The Reassure study is a longitudinal, community‐based study of Farsi and Dari speaking people who arrived in Australia since 2010, including: people with secure visa status (*Immigrants*: people from non‐refugee backgrounds and *Refugees*: people granted refugee protection with either secure (permanent visas) or semi‐secure (3–5 year visas) residency) and insecure visa status (*Asylum seekers*: people from refugee backgrounds with insecure residency (bridging visa)). The current paper reports on the findings of a cross‐sectional analysis of 12‐month data, the first time point when physical activity data were collected.

Recruitment was undertaken using a representative, multi‐stage time by location sampling frame applied across 12 randomly selected ethno‐specific grocery shops in Sydney, Australia. These shops are frequented by a wide range of newly arrived Iranian and Afghan community members to obtain traditional foods, enabling access to the community in a manner that minimised recruitment bias. Customers were invited based on the time they entered the shop (randomised across the study using probability proportional to size). A Kish grid approach was then applied to randomly select one member from consenting customers' family unit [33]. This helped to ensure that any adult member of a family (not necessarily the “shopper” in the family) was represented in the sample. Eligibility criteria were that participants were 18 years or over, spoke Farsi or Dari, identified Iran or Afghanistan as their country of birth, and had arrived in Australia since 2010. Participants were reimbursed $20 for the initial interview and $20 following each sixth month assessment. Ethics approval was obtained from the University Human Research Ethics Committee (HC16637).

### 2.2 Procedures

Initial data collection occurred in February 2017 with the 12‐month follow‐up in February 2018. The initial interview assessed a broad range of demographic and migration factors, including PTSD, depression, trauma history, postmigration stressors, and English language skills. Data on age, trauma count, and country of origin were collected at baseline. Data on physical activity, PTSD symptoms, depression symptoms, and current visa status were collected at the 12‐month assessment occasion. All interviews were conducted face‐to‐face or by phone by bi‐cultural researchers fluent in Farsi/Dari. Data was stored in deidentified format on secure servers.

### 2.3 Measures

#### 2.3.1 Demographics and Visa Status

Gender, country of origin (Iran, Afghanistan) and age were collected. Participants indicated their current visa status living in Australia as: (a) immigrants (e.g., citizenship, skilled, spouse, student); (b) refugees (those who were resettled under the Humanitarian intake programme, those who were granted permanent refugee status after arriving in Australia, and those granted temporary humanitarian protection visas); or (c) asylum seekers (people living on bridging visas while waiting for their asylum claims to be processed or those appealing a negative protection determination).

#### 2.3.2 Posttraumatic Stress Disorder and Trauma Events

The Harvard Trauma Questionnaire (HTQ) [34, 35] has been used extensively in international refugee populations [36, 37]. Participants indicated whether they had experienced any of the 16 listed traumatic events. These included items such as: torture, government harassment, death of a family member, being without medical treatment, physical assault, sexual assault, witnessing murder, kidnapping. Items were recoded into a dichotomous variable indicating whether the participant endorsed experiencing the event, except for witnessing murder, unnatural death or torture, in which case witnessing was coded as experiencing given the nature of these events. Lifetime exposure was calculated as a sum of yes responses. The HTQ includes 16 symptom items based on DSM‐IV PTSD criteria with response options ranging from: 1 (not at all) to 4 (extremely). Items included Recurrent thoughts or memories of the most hurtful or terrifying events; Feeling as though the event is happening again; A symptom severity score was calculated as an average score across all items. Cronbach's alpha was 0.96.

#### 2.3.3 Depression

The 15‐item Depression subscale from the Hopkins Symptom Checklist aims to measure the affective, cognitive, and somatic symptoms of depression (HSCL); [38]. It has been extensively used in conflict‐affected populations [39, 40]. Items included are *Feeling low in energy, slowed down; Crying easily; Feeling Sad; Feelings of worthlessness*. Response options range from1 (not at all) to 4 (extremely), and an average score is calculated. Cronbach's alpha was 0.94.

#### 2.3.4 Physical Activity

The Simple Physical Activity Questionnaire (SIMPAQ) [41] is a 5‐item clinical tool designed to assess physical activity among populations at high risk of sedentary behaviour. The tool measures a range of kinds of activity, including walking, moderate‐vigorous physical activity (MVPA), and work or chores based physical activity. Total time per week of MVPA was measured by asking participants about specific exercises or sports they had done in the last 7 days, and how long they did each one. The average number of minutes was then used to generate a dichotomous variable of whether participants met WHO physical activity guidelines of 150 min per week. Given the known tendency for people to underreport sedentary behaviour, sedentary time was calculated by subtracting the total reported physical activity per day (MVPA, walking and work) from the time awake per day.

### 2.4 Statistical Analyses

#### 2.4.1 Aims 1 and 2

Chi Square tests (physical activity guidelines) and one‐way Analysis of Variance (ANOVA) (sedentary behaviour) tested for associations with demographic variables. The intra‐cluster correlation coefficients were calculated for dependent variables to determine whether clustering by recruitment shop needed to be modelled in a mixed‐models analysis. Coefficients were less than one, so the effect of recruitment shop was deemed to be negligible, and one‐way ANOVA was used.

#### 2.4.2 Aim 3

Hierarchical logistic regression was used to test whether there was an independent association between visa status and achievement of the physical activity guidelines after controlling for age, gender, country of origin, and history of trauma. The controlling variables were entered in the first step with the dichotomous outcome of > 150 or < 150 min exercise per week. Hierarchical multiple regression was used to test for an independent association between insecure visa status and sedentary time after controlling for the same variables. Significance level was set at *p* = 0.05.

#### 2.4.3 Aim 4

**FIGURE 1:**
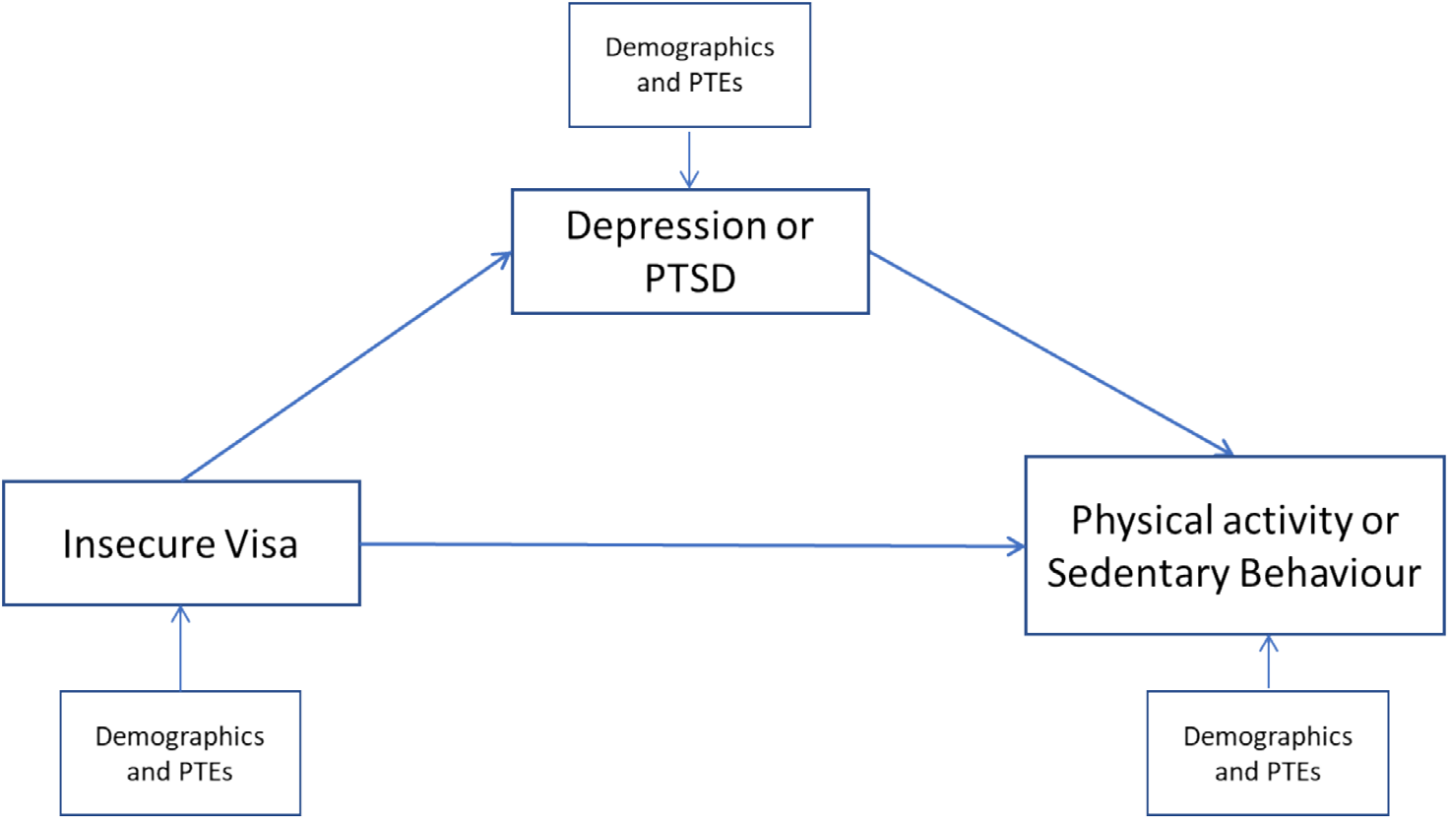
The impact of insecure visa status on meeting the physical activity guidelines is mediated by depression (Model 1) and PTSD symptoms (Model 2).

Path analysis was used to test a theoretical model that the association between visa status and physical activity would be mediated by psychiatric symptoms (depression or anxiety). See Figure 1. Separate models were tested in which the physical activity outcome (separate models for inactive/active and hours of sedentary time) was regressed on visa (secure/insecure) and symptoms (separate models for depression and posttraumatic symptoms). When achieving the physical activity guidelines was the dependent variable, this was declared as a categorical variable. Symptoms were then regressed on visa status, controlling for demographic variables (gender, age, country of origin and trauma count). Non‐significant paths were removed in an iterative process to improve model fit. In addition, an indirect path from visa to symptoms to physical activity tested for mediation effects. All path analyses were conducted in Mplus version 8.

## 3 Results

Data were collected from *n* = 276 Dari and Farsi speaking participants; mean age was 35 years (SD = 9.6). Sixty‐eight percent (*n* = 188) were males; 79% (*n* = 217) were from Iran; 36% (*n* = 98) had insecure visa status. People with insecure visas reported experiencing an average of 7.3 traumatic events (SD = 4.1) compared to people with secure visas who reported an average of 5.4 (SD = 4.3), *F*(2,272) = 13.9, *p* < 0.001. People with insecure visas (*M* = 33.3); (SD = 9) were also significantly younger than people with secure visas (*M* = 36.7; SD = 9.8), *F*(1,271) = 8.38, *p* = 0.004; more likely to be male (78%) compared to people with secure visas (62%), *Χ* ^2^ = 6.702, *p* = 0.01. There were no differences between visa status groups in country of origin.

### 3.1 Aim 1

In total, 39% (*n* = 108) of the sample achieved the WHO physical activity guidelines (see Table 1). There were no significant differences between males and females (*p* = 0.23). People from Iran were significantly more likely to meet the guidelines (*p* = 0.023). People with insecure visas were significantly less likely to report meeting the guidelines compared to people with secure visas (*p* = 0.001). Those who met the guidelines reported fewer categories of past traumatic events (mean = 5.4 (SD = 4.3) vs. 7.3 (SD 4.1), *p* < 0.001).

**TABLE 1:** Physical activity and sedentary time by gender, country, torture experiences and visa status.

	Moderate vigorous physical activity					Sedentary time (calculated)			
		< 150 min	> 150min	*Χ* ^2^	*p*	Mean	SD	*t*	*p*
Male	Count	119	69	1.46	0.227	16.6	1.4	3.5	**< 0.001**
	%	63%	37%						
Female	Count	49	39			16	1		
	%	56%	44%						
Iran	Count	124	93	5.17	**0.023**	16.3	1.3	3.57	**< 0.001**
	%	57%	43%						
Afghanistan	Count	42	15			17	1.2		
	%	74%	26%						
Insecure visa	Count	73	25	11.42	**0.001**	16.8	1.4	3.52	**0.001**
	%	75%	25%						
Secure visa	Count	94	81			16.3	1.2		
	%	54%	46%						

				*F*	*p*	Correlation	*p*
Trauma count	Mean	7.3	5.4	13.9	**< 0.001**	0.244	**< 0.001**
	SD	4.1	4.3				

*Note:* Bold indicates significant values.

### 3.2 Aim 2

Mean calculated sedentary time across the entire sample was 16.43 h (SD = 1.3). Females reported significantly less sedentary time per day compared to men (mean difference = 36 min, *p* = 0.001). People from Iran reported significantly less sedentary time than people from Afghanistan (mean difference 42 min, *p* < 0.001). People with insecure visas reported significantly more sedentary time (mean difference = 30 min, *p* = 0.001). Trauma count was significantly positively correlated with sedentary time (*r* = 0.24, *p* < 0.001). See Table 1.

### 3.3 Aim 3

Visa status showed a significant effect on whether participants met the PA guidelines after controlling variables, Wald = 12.3, *p* < 0.001. The odds of not meeting the physical activity guidelines were 3.06 times higher for people with insecure visas compared to those with secure visas. For sedentary time, visa status accounted for significant variance after controlling variables, Beta = 0.184, *p* = 0.003. See Table 2 for details.

**TABLE 2:** Logistic and linear regression analyses on physical activity guidelines and sedentary time.


	B	S.E.	Wald	df	Sig.	Exp(B)
Model 1	*Χ* ^2^ = 43.7, *p* < 0.001						
	Logistic regression on PA guidelines (150 min per week)						
	Age	−0.68	0.018	14.73	1	**< 0.001**	0.935
	Gender	0.169	0.308	0.3	1	0.582	1.184
	Country	0.948	0.369	6.61	1	**0.01**	2.580
	Trauma events	−0.073	0.035	4.26	1	**< 0.001**	0.93
Model 2	Change *Χ* ^2^ = 43.7, *p* < 0.001						
	Visa status	1.12	0.319	12.28	1	**< 0.001**	3.062


	*B*	S.E.	Beta	*t*	Sig.
Model 1	*F* = 13.091, *p* < 0.001					
	Linear regression on sedentary time					
	Age	0.014	0.008	0.1	1.71	0.08
	Gender	−0.311	0.172	−0.112	−1.81	0.071
	Country	0.607	0.187	0.192	3.25	**0.001**
	Trauma events	0.042	0.019	0.136	2.18	**0.03**
Model 2	*F* change = 9.065, *p* = 0.003					
	Visa status	0.499	0.166	0.184	3.011	**0.003**

*Note:* Bold indicates significant values.

### 3.4 Aim 4

*See* Table 3 for model fit statistics. For Models 1 and 2, In the first step, the categorical variable of whether participants met the activity guidelines was regressed on visa status. There was a direct effect of visa status on whether participants met the activity guidelines, standardised Beta = −0.632, *p* < 0.001. In step 2, this effect was mediated by depression symptoms, see Table 4, and PTSD symptoms. For Models 3 and 4, sedentary time was regressed on visa status. There was a direct effect of visa status on sedentary time, standardised Beta = 0.196, *p* = 0.001. In step 2, this effect was mediated by depression symptoms, see Table 4, and PTSD symptoms.

**TABLE 3:** Fit statistics for mediation path models.

Meeting the physical activity guidelines (150 min per week)		Sedentary behaviour (hours per day)	
Direct step		Direct model	
Step	Fit statistics	Step	Fit statistics
Step 1	Fit *Χ* ^2^ = 15.431, *p* = 0.017; RMSEA = 0.076; CFI = 0.720; TLI = 0.440; SRMR = 0.216	Step 1	*Χ* ^2^ = 2.399, *p* = 0.121; RMSEA = 0.071; CFI = 0.980; TLI = 0.859; SRMR = 0.031
Model 1 Depression mediation step		Model 3 Depression mediation step	
Step 2	*Χ* ^2^ = 4.254, *p* = 0.373; RMSEA = 0.015; CFI = 0.997; TLI = 0.990; SRMR = 0.075	Step 2	*Χ* ^2^ = 8.991, *p* = 0.11; RMSEA = 0.054; CFI = 0.989; TLI = 0.973; SRMR = 0.041
Model 2 PTSD mediation step		Model 4 PTSD mediation step	
Step 2	*Χ* ^2^ = 4.041, *p* = 0.4; RMSEA = 0.006; CFI = 0.999; TLI = 0.998; SRMR = 0.071	Step 2	*Χ* ^2^ = 12.77, *p* = 0.05; RMSEA = 0.064; CFI = 0.983; TLI = 0.965; SRMR = 0.045

**TABLE 4:** Path analyses of the impact of visa status and symptoms on physical activity outcomes.

Meeting the physical activity guidelines (150 min per week)							Sedentary behaviour (hours per day)						
	Beta	*p*	Covariates		Beta	*p*		Beta	*p*	Covariates		Beta	*p*
Model 1 depression symptoms							Model 3 depression symptoms						
Activity on depression symptoms	−0.483	< 0.001	Activity on	Age	−0.290	< 0.001	Sedentary on depression symptoms	0.379	< 0.001	Sedentary on	Country	0.219	< 0.001
				Country	−0.178	0.021							
Depression symptoms on insecure Visa	0.662	< 0.001	Depression symptoms on	Age	0.092	0.039	Depression symptoms on insecure visa	0.725	< 0.001	Depression symptoms on	Age	0.107	< 0.001
				Trauma count	0.224	0.01					Trauma Count	0.188	< 0.001
Indirect visa—depression—activity	−0.320	< 0.001	Insecure visa on	Age	−0.191	0.001	Indirect visa—depression—sedentary	0.275	< 0.001	Insecure visa on	Age	−0.197	< 0.001
				Trauma count	0.305	< 0.001					Trauma count	0.301	< 0.001
Model 2 PTSD symptoms							Model 4 PTSD symptoms						
Activity on PTSD symptoms	−0.426	< 0.001	Activity on	Age	−0.314	< 0.001	Sedentary on PTSD symptoms	0.374	< 0.001	Sedentary on	Country	0.696	< 0.001
				Country	−0.179	0.014							
PTSD symptoms on insecure visa	0.707	< 0.001	PTSD Symptoms on	Trauma count	0.242	0.005	PTSD Symptoms on Insecure Visa	0.725	< 0.001	PTSD symptoms on	Trauma count	0.04	< 0.001
				Age	0.041	0.347							
Indirect visa—PTSD—activity	−0.688	< 0.001	Insecure visa on	Age	−0.191	0.001	Indirect Visa—PTSD—sedentary	0.271	< 0.001	Insecure visa on	Age	−0.01	0.001
				Trauma count	0.305	< 0.001					Trauma count	0.034	< 0.001

## 4 Discussion

The primary finding of this study was that Farsi and Dari speaking asylum seekers with insecure residency were significantly less likely to achieve physical activity guidelines and engaged in more sedentary behaviour than compatriot refugees and immigrants who arrived in Australia over the same period with permanent or semi‐permanent residency. Forty‐six percent of Iranian and Afghan respondents with secure residency in Australia engaged in levels of moderate to vigorous PA that met the WHO recommendations [42]. In contrast, only 25% of Iranian and Afghan respondents with insecure residency achieved the WHO recommendations. Similar findings emerged when examining the mean sedentary time, with Iranian and Afghan respondents with insecure visas reporting high rates of sedentary time compared to those with permanent protection or residency visas. This pattern of higher levels of inactivity was evident after controlling for demographic factors and previous trauma exposure.

The significance of this finding should be contextualised with broader research linking physical inactivity with morbidity and mortality associated with noncommunicable diseases in the general population [11, 43]. These chronic health conditions are associated with reduced functioning, quality of life, and reduced life expectancy [44]. The path analyses indicate that mental health symptoms are a key factor impacting the relationship between insecure visa status and physical activity, extending understanding of the connection between restrictive immigration policies and mental health outcomes [45] to include associated lifestyle behaviours. Insecure residency is associated with a higher prevalence of lifestyle risk factors which are likely to lead to reduced quality of life and life expectancy for asylum seekers and to increased burden on the health system into the future. Since 2013, Australia's asylum policies have focused on strict border protection, marked by the introduction of Operation Sovereign Borders, offshore processing in Nauru and Manus Island, and the framing of asylum seekers as a security issue to deter unauthorised arrivals. Temporary Protection Visas (TPVs) and Safe Haven Enterprise Visas (SHEVs) allowed only temporary stays with limited rights, while fast‐track assessments and resettlement bans for boat arrivals further restricted pathways to permanency. Australia faced widespread criticism for human rights violations in detention centres and costly regional resettlement deals. Advocacy for reform persisted, and in 2023, the Labour government abolished TPVs and SHEVs, providing permanent residency pathways, though key restrictive policies like offshore processing and boat turn backs remain.

These findings add to a report released by Australia's Human Rights Commission, documenting the harmful psychosocial impacts on approximately 30 000 asylum seekers who lived with insecure residency for more than 5 years. These include reduced access to employment, education and social inclusion [11]. Providing refugees with secure residency is associated with large reductions in mental health symptoms [45]. Research in a nationally representative sample of children from refugee families with secure residency living in Australia found similar reported levels of emotional difficulties to children in the general Australian population [8]. This highlights how people from refugee backgrounds who are offered the settlement services available to those in Australia's humanitarian intake programme can recover and flourish. In contrast, our findings show that people who live indefinitely with insecure residency are at increased risk of mental distress and reduced physical activity, both of which are associated with morbidity and mortality.

It is important to consider the role of mental health symptoms in the relationship between insecure residency and physical health outcomes. A large longitudinal study of resettled refugees living in the UK [46] found that the prospective relationship between post‐migration living stressors and physical health outcomes was fully mediated by emotional distress. The current study helps to understand the mechanisms whereby a key social determinant of health (insecure residency) may impact on later health for this marginalised group. In addition, post‐migration stressors which interfere with health behaviours (such as language skills, access to health information, socio‐economic status and cultural factors) are likely to contribute to the observed levels of physical inactivity [47].

The results showed a significant difference in engagement with physical activity between people from Iran and Afghanistan. In the Iranian migrants' community, a confluence of cultural, societal norms, education, gender roles, migration patterns, and socioeconomic factors plays a critical role in promoting adherence to physical activity guidelines [48]. The country's tradition in physical activities, such as wrestling, coupled with societal norms that value fitness, fosters an environment that encourages and celebrates regular exercise. Enhanced by social support systems that promote group activities, these cultural aspects are bolstered by greater access to health education, which raises awareness about the benefits of physical activity. Gender norms in Iran provide women with more opportunities and acceptance to participate in physical activities, contrasting with more restrictive cultures [49]. Migration introduces diverse cultural influences, potentially altering physical activity patterns among migrants. Additionally, Iran's economic stability and the variances in socioeconomic status that influence access to physical activity resources [50] may further contribute to a higher engagement in sports and fitness activities compared to Afghanistan. Cultural considerations in promoting physical activity are therefore fundamental to ensuring safe, sustainable and efficacious interventions [51].

Given that previous evidence has shown that providing asylum seekers with secure residency leads to reductions in mental health symptoms [45], the provision of residency security is likely to lead to commensurate improvements in physical activity. However, the cross‐sectional nature of this study precludes the drawing of causal inferences. The relationship between mental health symptoms and post‐migration stressors is likely to be bi‐directional. In future waves of the current longitudinal study, relationships between depression and PTSD symptoms, post‐migration stressors and physical inactivity will be examined prospectively.

This study has a number of important implications for the field of health promotion. Firstly, our findings underscore the significant impact of insecure visa status on both physical activity levels and the mental health of refugees, immigrants, and asylum seekers, highlighting the need for targeted interventions [52] and reinforcing this population as a priority group for health promotion interventions. Secondly, results suggest that service providers should tailor programmes to address the unique challenges these groups face, including limited access to healthcare and uncertain living conditions. Finally, integrating mental health support with physical activity and health promotion initiatives is warranted given the potential contribution of each to protecting and promoting health outcomes. The findings advocate for policy reforms towards more secure residency statuses to improve health outcomes and emphasise the importance of including mental health specialists in these efforts [53]. Additionally, the research supports the development of public health initiatives that address barriers to physical activity specific to these vulnerable groups. By providing empirical evidence on the adverse effects of current immigration policies, the paper aims to inform policymakers and contribute to the re‐evaluation of policies that negatively impact the health of asylum seekers and refugees. It also warns of the long‐term health implications of residency insecurity, suggesting a potential future burden on the healthcare system [54, 55, 56]. Overall, the paper calls for comprehensive healthcare planning and policy development that considers the well‐being of refugees, immigrants, and asylum seekers, ensuring better health outcomes and overall well‐being for these communities in Australia.

## 5 Limitations

Visa status was used to determine whether participants were classified as immigrants or refugees. It is possible that some participants on non‐humanitarian visas may have come from a refugee background. We controlled for the number of potentially traumatic experiences to account for this. We compared people on bridging/rejected visas to all other visa types, which included people on *Temporary Protection Visas* (TPV) and *Safe Haven Enterprise Visas* (SHEV), both of which do not offer permanent protection. These visa categories also have harmful impacts on mental health [3, 45] however, for the purpose of this study, we used bridging visas as a marker for immediate danger of repatriation. In addition, people on TPV and SHEV visas have greater access to employment, education, and health care, making their experience of postmigration living stressors distinct from people on bridging/rejected visas. This study was also limited by self‐report measures, which may overestimate mental health symptoms compared to diagnostic assessments and which are less accurate than objective measures of physical activity. Finally, cultural considerations relating to the definition of PA and the potential influence of different PA domains (occupational, transport, leisure‐time and household activity) were not able to be captured in these data. Future research should consider the impact of different PA domains.

## 6 Future Research

To effectively support the mental health and well‐being of asylum seekers, future research efforts should focus on understanding the potential long‐term benefits of physical activity as a coping strategy. Physical activity is a powerful tool in promoting mental health among different populations [14]. It is associated with reduced levels of stress, anxiety, and depression while increasing self‐esteem and overall well‐being. However, the unique circumstances of refugees, such as limited access to resources and social support networks, may affect their ability to engage in physical activity as a means of coping [16]. Therefore, comprehensive studies should be conducted to investigate the feasibility and effectiveness of combining physical activity programmes tailored to the specific needs and limitations of asylum seekers in Australia. By recognising physical activity as a potential coping strategy, policymakers and organisations can develop targeted initiatives that not only reduce mental health challenges but also promote social integration and resilience among asylum seekers in Australia. Ultimately, this research can contribute to more inclusive and supportive policies and programmes for asylum seekers, acknowledging the importance of their psychological well‐being as they navigate the uncertain path to asylum and building a new life in a foreign land.

## 7 Conclusion

The mental health of refugees and asylum seekers deteriorates as a consequence of prolonged exposure to residency insecurity. We found that the health promoting behaviour of physical activity is also compromised among this group. Current restrictive immigration policies may lead to severe adverse health outcomes in the future.

## Conflicts of Interest

The authors declare no conflicts of interest.

## Data Availability

Data available on request due to privacy/ethical restrictions. The data that support the findings of this study are available on request from the corresponding author. The data are not publicly available due to restrictions [e.g., their containing information that could compromise the privacy of research participants].
